# A Rare Case of Rectosigmoid Small Cell Carcinoma

**DOI:** 10.7759/cureus.41339

**Published:** 2023-07-03

**Authors:** Zhuang Hui Mark Le, William Swee Keong Khoo, Kaushik Kumar

**Affiliations:** 1 Department of General Surgery, Logan Hospital, Brisbane, AUS; 2 Department of General Surgery, Redland Hospital, Brisbane, AUS; 3 Department of General Surgery, Queen Elizabeth II Hospital, Brisbane, AUS

**Keywords:** colorectal neuroendocrine malignancy, extrapulmonary small cell carcinoma, general surgery, colorectal small cell carcinoma, colorectal cancer

## Abstract

Colorectal small cell carcinomas are very rare neuroendocrine malignancies of the colon or rectum. They have poor prognosis due to the aggressive and highly recurrent nature of the disease. It is a malignancy that is also poorly understood with limited literature, and thus there is no consensus in management. This case report presents the clinical features and radiological images of an otherwise healthy 74-year-old gentleman with a rare and aggressive 112-mm rectosigmoid small cell carcinoma with evidence of metastatic disease. This report will also discuss the most current and pertinent diagnostic and therapeutic recommendations from the literature.

## Introduction

Colorectal small cell carcinomas (CSCCs) are very rare, aggressive, and highly recurrent neuroendocrine malignancies of the colon or rectum [1]. CSCCs account for only 0.2% of colorectal carcinomas (CRCs) and have a poor prognosis [2-4]. Furthermore, there are a limited number of published cases of CSCCs, and even fewer articles on the prognostic outcomes of the various approaches to treating CSCCs (e.g., the role of neoadjuvant versus adjuvant radiotherapy and chemotherapy). As such, there is currently no consensus in the management of CSCCs. This case report presents the clinical progression and radiological images of a 74-year-old gentleman with a rare and large 112-mm centrally necrosed rectosigmoid mass involving the porta hepatic lymphatics. The pertinent clinical features, and the diagnostic and current therapeutic recommendations from the literature will also be discussed.

## Case presentation

**Figure 1:**
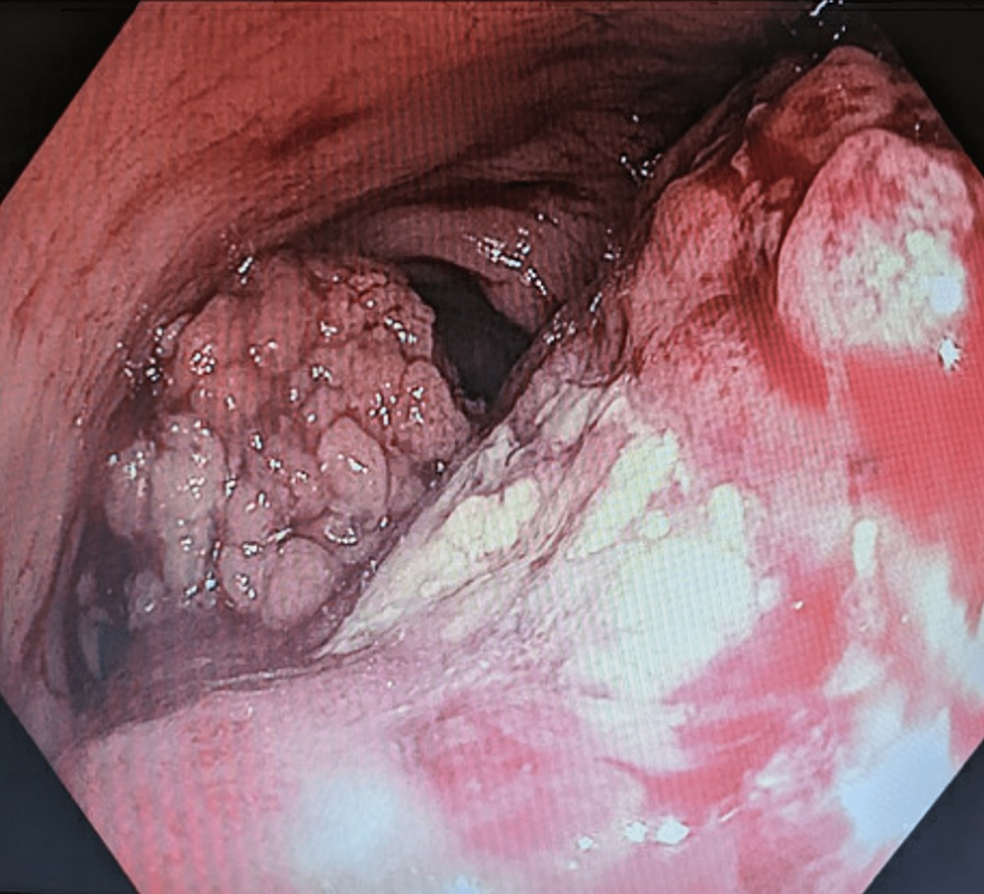
Endoscopic image showing the 9-cm fungating, circumferential, and partially obstructing mass at the rectosigmoid junction.

A 74-year-old man was referred to the colorectal clinical by his general practitioner with a six-month history of lower abdominal pain, loose bowel motions, bleeding per rectum, and unintentional weight loss. He underwent a computer topography (CT) one month prior to clinic review, which showed a focal 89-mm partially necrotic pelvic mass. The patient had no past medical history. The only surgical history was a bilateral laparoscopic inguinal hernia repair with mesh. There was no family history of colorectal cancer, and he has had no colonoscopies in the past. He was an active smoker of 40 pack-years. The only pertinent finding on physical examination was suprapubic abdominal tenderness. Hematological investigations showed a carcinoembryonic antigen (CEA) of <1.0 mg/mL (<5.0 mg/mL) and chromogranin A of 454 ug/L (20-102 ug/L). An urgent colonoscopy demonstrated a 9-cm fungating, circumferential, and partially obstructing mass approximately 8 cm from the anal verge (Figure 1). Histological sections revealed neoplastic infiltrate, nuclei smearing, and foci of nuclear molding, features favoring small cell carcinoma. Immunohistochemistry was positive for synaptophysin and insulinoma-associated protein 1 (INSM1).

**Figure 2:**
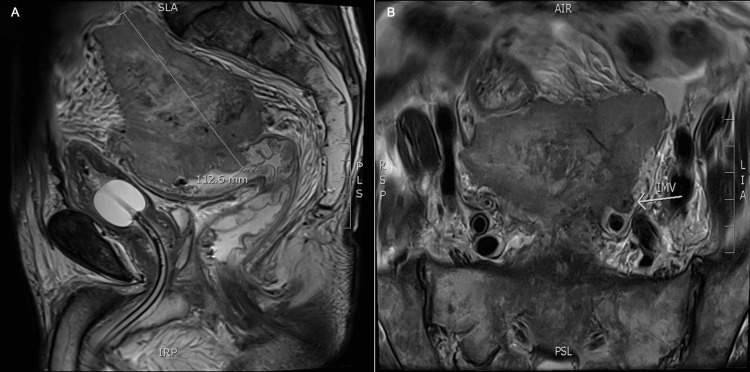
MRI demonstrating a 112-mm rectosigmoid tumor with direct invasion of the presacral fascia above the anterior peritoneal reflection, direct invasion of IMV, and a 5-mm left obturator lymph node (A = sagittal view, B = axial view). MRI, magnetic resonance imaging; IMV, inferior mesenteric vein

Unfortunately, two weeks from initial review and two days before planned surgical resection of the rectal malignancy, the patient presented to the emergency department in uroseptic shock requiring urgent ureteric stent insertion and intensive care unit admission for escalating vasopressor support. He did not have any bowel-related obstructive symptoms at this time. A repeat CT performed on presentation to the emergency department demonstrated enlargement of the now 112-mm centrally necrosed rectosigmoid mass with left distal ureteric involvement and a new 12-mm porta hepatic lymph node. Inpatient magnetic resonance imaging (MRI) demonstrated the same rectosigmoid tumor with direct invasion of the presacral fascia above the anterior peritoneal reflection, direct invasion of the inferior mesenteric vein, and a 5-mm left obturator lymph node (Figure 2). Positron emission tomography (PET) staging demonstrated a fluorodeoxyglucose (FDG)-avid rectosigmoid mass, FDG-avid right vesico-ureteric junction lymph node, and small non-FDG avid 3-mm right lung nodule (T4aN1M1).

A multidisciplinary approach was taken, and the patient was consequently referred to medical oncology for neoadjuvant chemotherapy and planned anterior resection of the rectum with creation of loop ileostomy. However, due to the rapid deterioration of health in the context of the ICU admission for uroseptic shock, the patient and his family later decided against radical surgical resection and opted for palliative transition instead. He was discharged home to comfort care. No palliative radiotherapy or chemotherapy was undertaken.

## Discussion

CSCCs are very rare and poorly understood extrapulmonary neuroendocrine carcinomas of the colon or rectum [1]. They represent 10% of small cell carcinomas (SCCs) and only 0.2% of CRCs [2-4]. The median age of onset is 55 years with male predominance [2]. CSCCs are aggressive malignancies and have very poor prognosis due to high recurrence rates, with a median survival for limited disease of 10 months and for metastatic disease of 4 months, as reported by multiple studies [1,2,4-6]. The pathophysiology of CSCCs is unknown, although some studies suggest an association with tubulovillous adenomas [7,8].

CSCCs usually manifest asymptomatically; however, non-specific abdominal features such as abdominal pain, per rectum bleeding, changes in bowel movements, and constitutional symptoms have been reported [3,4]. Features of liver or skeletal dysfunction may also be present (common sites of metastases via lymphatic and hematological spread, respectively). CSCCs are diagnosed histologically with immunohistochemical staining via biopsies obtained endoscopically. The histopathological features of CSCCs are identical to pulmonary small cell carcinomas: spindle-shaped cells with hyperchromatic nuclei, reduced cytoplasm, and multiple mitoses [2,3]. Immunohistochemical staining is positive for CD56, synaptophysin, and chromogranin A [1]. Additionally, the presence of at least two positive neuroendocrine tumor markers (e.g., synaptophysin, chromogranin A, and neuron-specific enolase) is supportive for the diagnosis of SCCs [2]. The immunohistochemistry for this case was positive for synaptophysin and insulinoma-associated protein 1 (INSM1).

Currently, there is no consensus on the optimal treatment for CSCCs due to poor prognosis and high recurrence of disease. Most therapeutic suggestions are extrapolated, due to limited evidence, from the more common pulmonary variation of SCC [1,3]. There is also no dedicated staging system for CSCCs. As such, approach to management is currently guided by whether patients present with limited or extensive disease (defined as not amenable to radiotherapy) [1-4]. For patients with limited disease, multiple studies suggest definitive surgical resection with aggressive radiotherapy and adjuvant multidrug chemotherapy (e.g., etoposide or irinotecan, and platinum agent) [1-3,8,9]. Fitzhugh et al. and Elias et al. suggested neoadjuvant chemotherapy followed by resection with subsequent adjuvant chemoradiotherapy [3,4]. The prognostic benefits associated with these approaches are, however, still unknown given that so few cases achieve long-term survival. Zhou et al. suggested that a combination of systemic chemotherapy and radiotherapy was as effective as radical surgical resection [10]. Horn et al. further proposed the use of immunotherapy (e.g., atezolizumab) as an additional medical therapy [11]. On the other hand, for patients with extensive CSCC disease, literature consensus is supportive care and palliative chemotherapy [1-3,8,9]. However, regardless of current treatment modalities, prognosis of CSCCs remains poor.

## Conclusions

In conclusion, CSCCs are very rare, difficult to clinically diagnose, and aggressive colorectal malignancies with no prognostically significant treatment. There is no consensus on management. Available literature on CSCCs is also heterogenous and mostly in the form of case reports. This case report highlights the importance of early diagnosis and a multidisciplinary approach to holistic and patient-centered treatment. Given the rarity of the disease and limited research, an international registry should be created to collect data on cases of CSCCs with the aim to improve diagnostic and therapeutic outcomes.
